# Role of topical antibiotic ointment in the lateral graft following underlay myringoplasty: a prospective randomised study

**DOI:** 10.1186/s40463-023-00674-1

**Published:** 2023-12-13

**Authors:** Zhengcai Lou, Zihan Lou, Tian Lv, Zhengnong Chen

**Affiliations:** 1 Department of Otorhinolaryngology, Yiwu Central Hospital, 699 Jiangdong Road, Yiwu City, 322000 Zhejiang Provice China; 2 Department of Otolaryngology-Head and Neck Surgery, Shanghai Sixth People’s Hospital Affiliated to Shanghai Jiao Tong University School of Medicine, Shanghai, 200233 China; 3 Department of Otolaryngology Head and Neck Surgery & Center of Sleep Medicine, Shanghai Sixth People’s Hospital Affiliated to Shanghai Jiao Tong University School of Medicine, Yishan Road 600, Shanghai, 200233 China; 4 Otolaryngological Institute of Shanghai Jiao Tong University, Yishan Road 600, Shanghai, 200233 China; 5 Shanghai Key Lab Sleep Disordered Breathing, Yishan Road 600, Shanghai, 200233 China; 6 Department of Otorhinolaryngology, Shanghai Jiao Tong University Affiliated Sixth People’s Hospital, Shanghai, 200233 China

**Keywords:** Myringoplasty, Antibiotic ointment, Middle ear infection, Graft

## Abstract

**Objective:**

The objective of this study was to compare the graft outcome and postoperative infection of with and without the use of antibiotic ointment following myringoplasty for the treatment of chronic perforations.

**Study Design:**

Randomized controlled trial.

**Material and Methods:**

135 chronic perforations were prospectively randomized to use of antibiotic ointment group (UAO, n = 68) or no use of antibiotic ointment group (NAO, n = 67) following myringoplasty. The graft outcomes and postoperative infection were compared among two groups at 6 months.

**Results:**

At postoperative 6 months, the graft infection rate was 4.4% in the UAO group and 10.4% in the NAO group, the difference was not significant (*P* = 0.312).The graft success rates were 92.6% in the UAO group and 91.0% in the NAO group, the difference was not significant (*P* = 0.979). In the UAO group, 3 patients with purulence ear discharge resulted in a residual perforation although they received ofloxacin ear drops and intravenous antibiotic therapy treatment. In the NAO group, 6 patients with purulence ear discharge resulted in a residual perforation, only one, with mild purulence discharge was successfully treated and closed. In addition, no significant between-group differences were observed pre- (*P* = 0.746) or post- (*P* = 0.521) operative air bone gap (ABG) values or mean ABG gain (*P* = 0.745). However, granular myringitis with minimal moistness but without infection has been noted in 3 (4.4%) patients in the UAO group and in 5 (7.5%) in the NAO group, the difference was not significant (*P* = 0.699).

**Conclusions:**

Use and non-use of antibiotic ointments for lateral packing of graft are both comparable methods following myringoplasty for postoperative infection and graft outcomes.

## Introduction

Myringoplasty has been successfully used to repair vairous perforation. Packing of the external auditory canal (EAC) after surgery is an established practice in most hospitals, this is believed to protect the operated site, hold the graft in situ [1, 2]. The type of packing varies between departments, with a wide range of individual preference based more on tradition than evidence [1, 2]. Some recent studies demonstrated that the type of EAC packing did not affect the graft outcome [3], which avoids the discomfort of ear pack removal and an unnecessary out-patient visit, thereby potentially reducing the cost of care. However, although biological materials packing is gradually reduced in the EAC packing, even it was abandoned entirely in some studies [2, 4], it is relatively common for surgeons to use antibiotic ointment only in EAC packing in tympanoplasty/tympanomastoidectomy [2, 3, 5],this is believed to prevent the infection of graft. Nevertheless, the antibiotic ointment may also adhere to the graft, ear canal or, in particular, the tympanomeatal flap, traumatizing these and disturbing the graft on removal [1, 2]. However, to the authors’knowledge, the fundamental need for the use of antibiotic ointment has not been established, there is little data on any difference of graft outcome and postoperative infection. The objective of this study was to compare the graft outcome, postoperative infection, and complications of with and without the use of antibiotic ointment in underly myringoplasty with no EAC packing of biological materials for the treatment of chronic perforations.

## Materials and methods

### Ethical considerations

This prospective Randomized Controlled Trial (RCT) was reviewed and approved by the Medical Ethical Committee of Yiwu central hospital. Informed consent was obtained from all participants.

### Methods

Study subjects were recruited from consecutive adult patients diagnosed with chronic perforation who visited the Department of Otorhinolaryngology, Head and Neck Surgery. The inclusion criteria was included: no marginal small-or medium size perforation with no involving the malleus umbo; dry ear at least 3 months prior to surgery based on patient history, good pneumatization of the middle ear and mastoid region, as shown by high-resolution computed tomography (HRCT). Prior to enrollment, HRCT was performed, and audiograms were recorded in all patients. Exclusion criteria were active inflammation during the 3 months prior to surgery, revision surgery, cholesteatoma, and requiring ossiculoplasty. The perforation size was categorised as small (< 1/8 of the TM area), medium (1/8–1/4 of the TM area) [6, 7]. The following data were recorded: age, sex, side, perforation size, duration, myringosclerosis, and smoking status. Some studies demonstrated that was smoking status was significant prognostic factors positively influencing the success rate of myringoplasty [8, 9], thus, smoking status were included in our data.

Audiometric data were obtained preoperatively and 6 months postoperatively. Pure-tone averages (PTAs) were calculated for both air conduction (AC) and bone conduction (BC) by averaging the thresholds at 500, 1000, 2000, and 3000 Hz; in most cases, a threshold of 4000 Hz was used to interpolate the threshold of 3000 Hz per the standards of the Hearing Committee of the American Academy of Otolaryngology-Head and Neck Surgery. Pre- and postoperative air bone gap (ABG) was calculated by subtracting the AC PTA from the BC PTA. ABG closure was calculated as preoperative ABG minus postoperative ABG [3].

### Randomization and blinding

Randomization was performed using computer-generated block randomization. Patients were randomized into 2 treatment groups: use of antibiotic ointment in the EAC (UAO group) and no use of antibiotic ointment (NAO) group. The allocation was performed using a sealed envelope system. All the patients accepted endoscopic cartilage underlay myringoplasty by the same surgeon under total intravenous anesthesia, which does not involve the raising tympanomeatal flap.The participants were unmasked to the treatment allocation.

### Surgical technique

All patients underwent endoscopic perichondrium-cartilage underlay myringoplasty without tympanomeatal flap elevation.The perforation edges were freshened to de-epithelialize and encourage migration of the mucosal layer and epithelium.

A single-layer, perichondrial, composite cartilage graft was harvested from the ipsilateral tragus. The lateral perichondrium was peeled circumferentially, with the pedicle attachment the cartilage graft. The cartilage graft was shaped to be 1 mm larger than the perforation margins, with the perichondrium more than 1–2 mm the cartilage graft.

The middle ear was packed with Biodegradable NasoPore (Biodegradable NasoPore, Stryker Canada, Hamilton, ON, Canada. Biodegradable NasoPore is a biodegradable synthetic polyurethane foam, the expected time for the material to dissolve is four weeks according to the manufacturer). The perichondrium-cartilage composite graft was placed trans-perforation, the cartilage graft was placed medial to the remnant TM and the annulus. Also, the perichondrium was placed medial to the remnant TM and the annulus.The cartilaginous was then packed with antibiotic-soaked gauze, up to the tragus incision.

### Use of antibiotic ointment (UAO) group

Erythromycin ointment was injected into the lateral surface of the graft and the TM remnant using a sterile 14-Gauge needle.

### No use of antibiotic ointment (NAO) group

No any was applied on the lateral surface of the graft and the TM remnant.

### Postoperative follow-up

Patients were discharged on the day after surgery. Postoperative follow-up was scheduled at weeks 1, 2 and 4 and at months 3, and 6. At each follow-up time point, the graft status were endoscopically examined, and the patients were asked about altered taste, vertigo, and tinnitus. Audiometric evaluations were performed at 6 months postoperatively. All postoperative complications were recorded, including graft infection, graft lateralization, significant blunting or medialization.

### Outcome measures

The primary outcome measures were the graft success and audiometric outcomes; secondary outcome measures were graft infection, graft lateralization, significant blunting or medialization. Graft success was defined as an intact graft without residual or re-perforations. Graft infection was defined as purulence discharge in the middle ear and EAC resulting in graft absorb or graft necrosis.

### Statistical analyses

Statistical analyses were performed with the aid of SPSS ver. 21 (SPSS Inc., IBM, Chicago, IL). Data are expressed as means (with standard deviations [SDs]) or percentages (%). The chi-squared test was used to compare categorical data. The Wilcoxon and Mann–Whitney U-tests were employed to compare non-parametric variables and the independent and paired samples t-tests were used to compare parametric variables. A *P*-value < 0.05 was considered to indicate statistical significance.

## Results

### Demographic characteristics

**Fig. 1:**
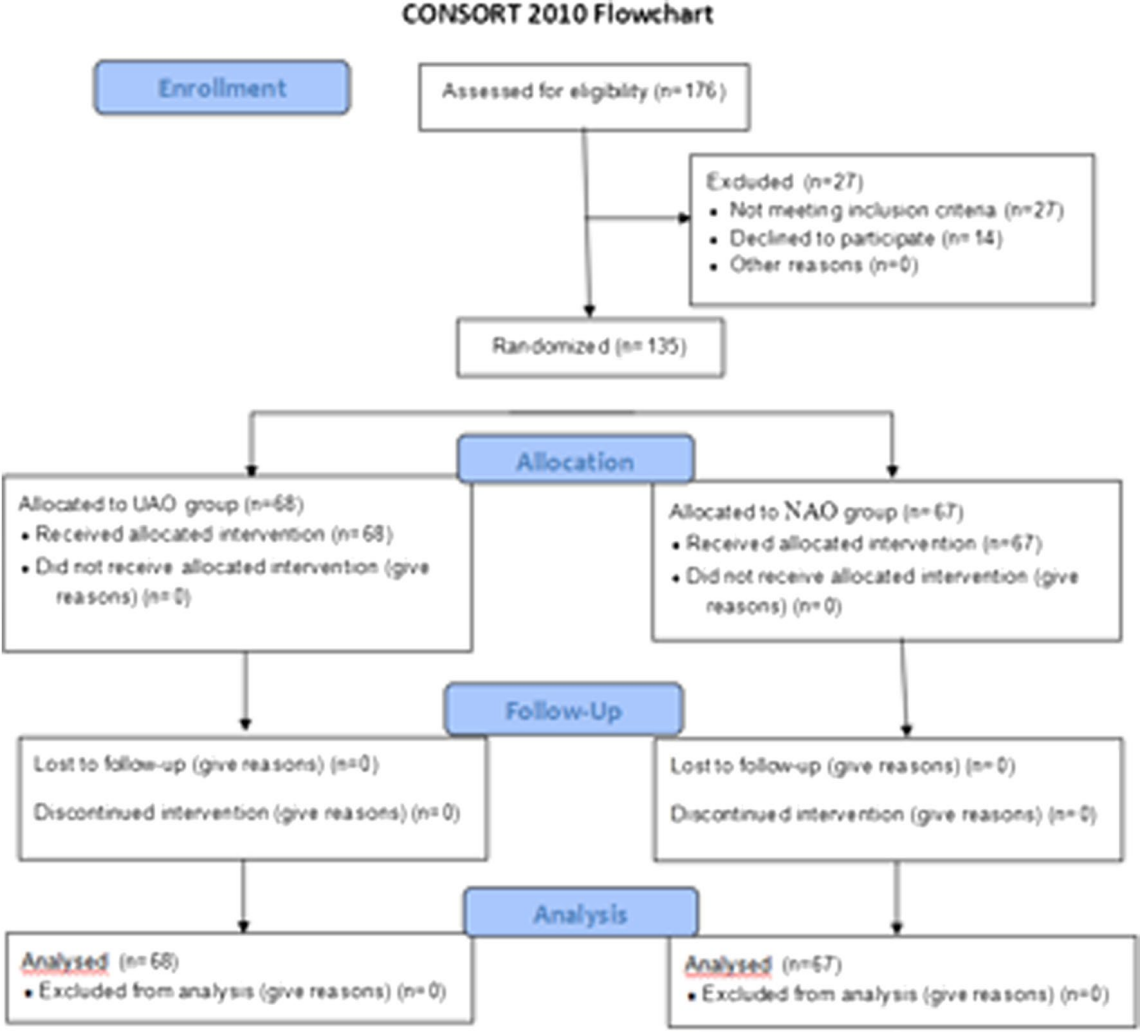
Enrollment, randomization, and follow-up of study participants

A total of 135 chronic perforations were included. Of the 135 ears, 68 perforations were in the UAO group and 67 perforations in the NAO group (Fig. 1). Demographic data are shown in Table 1. Sex, mean age, side, duration,perforation size, myringosclerosis, and smoking status were matched between the groups. All patients completed 6 months of follow-up.

**Table 1:** Demographic characteristic of patients

	UAO group (n = 68)	NAO group (n = 67)	*P* value
Sex (F:M)	29:39	26:41	0.780^a^
Age (years)	46.2 ± 3.1	47.0 ± 1.1	0.695^b^
Side of ear (L:R)	41:17	52:15	0.497^a^
Myringosclerosis (Y:N)	9:59	12:55	0.608^a^
Smoking status (Y:N)	7:61	9:58	0.766^a^
Duration,years	16.1 ± 2.4	15.9 ± 6.3	0.794^b^
Perforation size (S:M)	14:54	17:50	0648^a^
Graft infection, n(%)	3 (4.4%)	7 (10.4%)	0.312^a^
Residual perforation, n(%)	2 (0.0%)	0 (0.0%)	0.482^a^
Graft success, n(%)	63 (92.6%)	61(91.0%)	0.979^a^

^a^Chi-square test

^b^Independent Samples t-test

F: Female; M: Male; L: Left; R: Right

### Graft success

At postoperative 6 months, in the UAO group, residual perforation was seen in 5 patients. Of the 5 patients, 3 patients presented as purulence ear discharge at postoperative 7 days and was subsequently found to have failed, resulting in a residual perforation although none had persistent purulence discharge after ofloxacin ear drops and intravenous antibiotic therapy treatment, while middle ear infection was not found in the remaining 2 patients.

In the NAO group, 6 patients presented as purulence ear discharge at postoperative one week, who were subsequently found to have failed, resulting in a residual perforation, although purulence ear discharge was successfully treated with ofloxacin ear drops and intravenous antibiotic therapy treatment. In only one, mild purulence discharge was observed at postoperative 3 days and successfully treated with intravenous antibiotic therapy treatment for one week, the graft subsequently survived and the perforation was successfully closed.

**Fig. 2:**
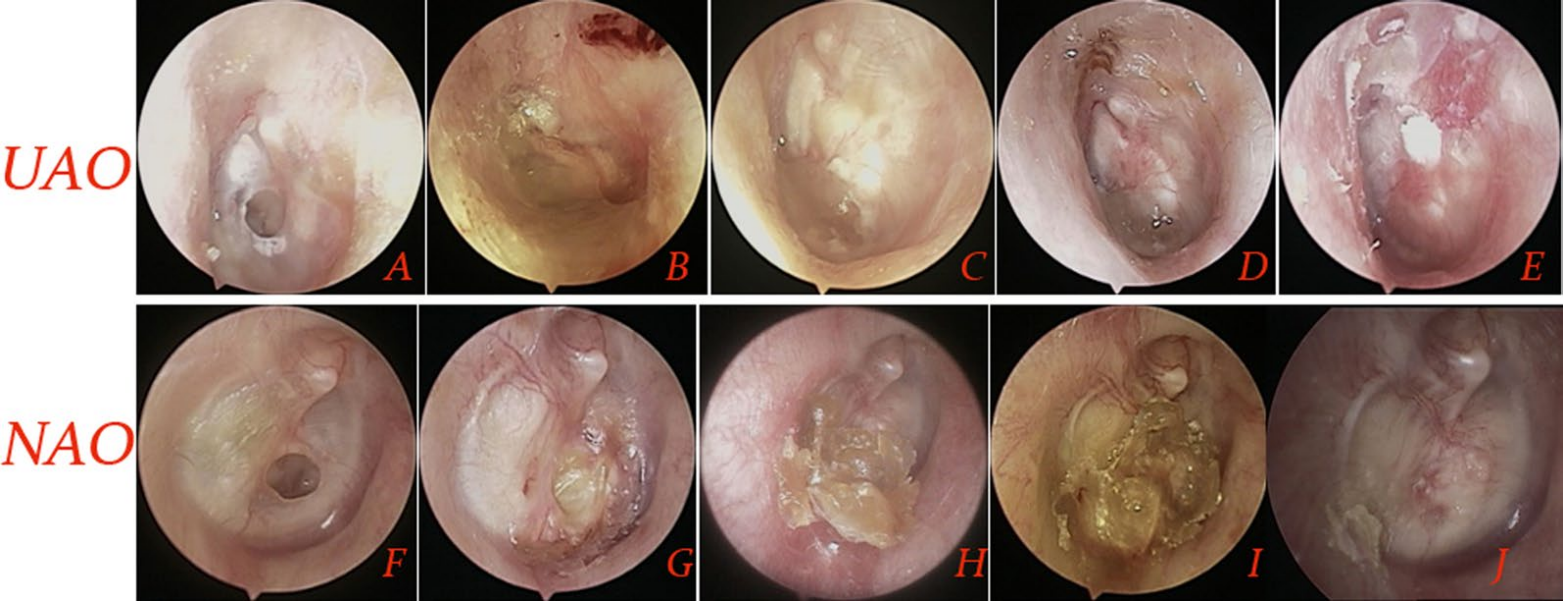
Followup procedure of two techniques. UAO technique. **A**: Preoperative perforations; **B**: one week after surgery; **C**: 4 weeks; **D**: 2 months; **E**:4 months. NAO technique. **F**: Preoperative perforations; **G**: 2 weeks after surgery; **H**: 4 weeks; **I**: 2 months and **J**: 3 months

Non-purulent exudate of varying degrees was seen in both groups at postoperative 1–4 weeks, except for the perforations with graft infection. The graft success rates were 92.6% (63/68) in the UAO group and 91.0% (61/67) in the NAO group, the difference was not significant (*P* = 0.979) (Fig. 2). The graft infection rate was 4.4% (3/68) in the UAO group and 10.4% (7/67) in the NAO group, the difference was not significant (*P* = 0.312).

### Hearing outcome

The postoperative ABG showed significant improvement compared to pre-operation in both groups, while no significant difference was observed in any group with regard to pre- and post-operation average BC PTA (Table 2). In addition, no significant between-group differences were observed pre- (*P* = 0.746) or post- (*P* = 0.521) operative ABG values or mean ABG gain (*P* = 0.745). Also, no significant between-group differences were observed pre- (*P* = 0.617) or post- (*P* = 0.639) operative mean BC PTA.

**Table 2:** Comparison of hearing gains, the air–bone gap, and bone conduction

	Pre-operative (dB ± S.D.)	Post-operative (dB ± S.D.)	*P*^a^	Gain (mean)	*P*^b^
UAO group (n = 68)					
Average ABG, dB	23.8 ± 2.6	10.4 ± 3.2	0.001*	13.5 ± 2.6	
Average BC PTA, dB	14.1 ± 2.8	13.9 ± 5.1	0.794		
NAO group (n = 67)					
Average ABG, dB	24.1 ± 1.7	11.2 ± 4.4	0.001*	13.1 ± 4.9	0.745
Average BC PTA, dB	14.5 ± 1.9	14.0 ± 2.8	0.681		

dB decibel, S.D. Standard deviation, ^a^Paired Samples test ^b^Mann Whitney U test

^*^P < 0.01

^a^Comparison between the same groups in regard to ABG or bone conduction, pre- and postoperatively

^b^Comparison between two groups in terms of gain, pre- or postoperatively

BC: bone conduction; PTA:pure-tone average;

### Complications

During followup period, no complications were observed in both groups, including deteriorative sensorineural hearing loss, altered taste, vertigo, or tinnitus. Also, no graft lateralization, significant blunting or medialization were found in any group. All the patients didn’t develop postoperative otitis media effusion and graft retraction. However, granular myringitis with minimal moistness but without infection has been noted in 3 (4.4%) patients in the UAO group and in 5 (7.5%) in the NAO group, the difference was not significant (*P* = 0.699).

## Discussion

It is traditional practice to place a dressing pack within the EAC following myringoplasty, the pack produces a temporary conductive hearing loss until removed. In addition, post-operative ear pack removal is a significant source of anxiety and discomfort, especially in the paediatric age group [2]. However, we have been unable to establish the rationale, or evidence base, for this practice. In fact, there is a paucity of work published on ear packing, except for comparisons of various types of ointment used with the packs. Nevertheless, recent some evidences suggested that the type of EAC packing did not affect the graft outcomes and increase the complications [3, 10].This avoids the discomfort of ear pack removal and an unnecessary out-patient visit, thereby potentially reducing the cost of care. Unfortunately, it is relatively common for surgeons to use antibiotic ointment only in EAC packing, this is believed to prevent the infection of graft [2, 3, 5]. However, the fundamental need for preveting graft infection via use of antibiotic ointment has not been established.

Some scholars recommended that topical antibiotic ointments are effective to use on open wounds in preventing wound infections [11]. However, although the graft infection rate in the NAO group was high than that of UAO group, it was not significantly different among two groups (10.4% vs 4.4%, *P* = 0.312). Thus, there was no documented evidence of the use of antibiotic ointment affecting graft infection following myringoplasty. This result was similar to previous findings. Hirschmann et al. [12] emphasized that topical antibiotic ointments is ineffective to prevent postoperative wound infections either for dermatologic surgery. Kamath et al. [13] found that there was reduction in the incidence of wound infection with the use of topical antibiotic ointment following surgeries for hip fractures. However, this was not statistically significant to recommend its use in routine practice (relative risk 0.430, 95% confidence interval (CI) 0.120–1.544). Dixon et al. [14] compared the effect of applying ointment to a wound and with no ointment, they found no significant differences in infection rate for all endpoints evaluated (2.3% vs. 1.4%, *P* = 0.490) and putting ointment on a surgical wound does not benefit the patient. Kalmeijer et al. [15] performed a double-blind, randomized, placebo-controlled study to determine whether use of mupirocin nasal ointment for perioperative eradication of Staphylococcus aureus nasal carriage is effective in preventing the development of surgical site infections (SSIs) and demonstrated that mupirocin nasal ointment did not reduce the SSI rate.

In the field of otologists, Cunningham et al. [16] applied unilaterally coating one grommet with antibiotic ointment to prevent the infection but the contralateral tube was not in 128 children undergoing bilateral tympanostomy tube placement, the incidence of postoperative infection was 4.7%. in untreated grommets and 2.3% in treated side, they believed that the technique of antibiotic ointment application to the tympanostomy tube at the time of surgery was not sufficiently efficacious to prevent infection. Meghji et al. [5] compared postoperation complications rate of the EAC packing using chloramphenicol ointment and packing using bismuth iodoform paraffin paste following tympanoplasty and found both comparable methods. In addition, no use of antibiotic ointment may reduce postoperative symptoms, fullness, and pressure.

In this study, Erythromycin ointment was choosed to pack lateral surface of the graft. Most of literatures reported that Staphylococcus aureus and Pseudomonas aeruginosa were the commonest bacteria for chronic suppurative otitis media (CSOM) [17–24], however, these most common bacteria showed the conflict results for susceptibility and resistance to various antibiotics [18–24]. Some studies found that Staphylococcus aureus showed high sensitivity to erythromycin [18–20] but only a few reported poor susceptibility [21, 22]. Erythromycin ointment was more useful in our department and in otologic clinc in other hospital [23, 24]. Unfortunately, bacterial culture and antimicrobial susceptibility testing were not performed for the patients with purulence discharge in both group in this study.

Although some scholars reported that the graft success rates following myringoplasty was not affected by the presence of preoperative otorrhea [17, 25], postoperative infection increase the flap and free graft necrosis [11, 26, 27]. In this study, the graft became partial or total necrosis in 9/10 patients with middle ear infection. The postoperative infection may affect the graft neovascularization, thereby result in the graft necrosis and absorption, increase the failure rate of graft. Previous study showed that the graft success rate depends principally on the absence of postoperative infection, placement of an adequately sized graft, no graft movement postoperatively, and the absence of any unrecognized middle ear mucosal disease [3]. In this study, the graft success rate was similar among two groups (92.6% vs. 91.0% (61/67). Of the 11 patients with graft failure, only 2 patients were attributed to the technique factor or the insufficient cartilage graft, while 9 were largely due to postoperative middle ear infection with a secondary partial or total necrosis of cartilage graft, thereby resulted in the residual perforation. The results agrees with previous studies. Yamamoto et al. [28] in the histologic study of homograft cartilages implanted in the middle ear found that there was partial absorption of cartilage when inflammatory changes occurred in the middle ear. Mintz et al. [29] observed that perichondritis and cartilage necrosis may result from infection introduced by repeated laryngeal manipulation. In addition, Elwany et al. [26] in the histochemical study of cartilage autografts in tympanoplasty found that the middle ear infection had a strong lethal effect on chondrocytes, and that cartilage grafts survive better in dry ears. They found that that partial or total necrosis of the graft was associated most commonly with Pseudomonas Aeruginosa, B.Hemolytic Stryptococci and Staphylococcus Aureus [26]. However, Staphylococcus Aureus has identified α-toxin as the primary agent causing the rapid death of cartilage cells (chondrocytes) [27]. Fortunately, in the NAO group, mild purulence discharge was observed in one patient at postoperative 3 days and treated with intravenous antibiotic therapy treatment, the graft subsequently survived and the perforation was successfully closed. Thus, it is crucial to early monitor the graft change and early application of antibiotic therapy treatment with mild purulence discharge for graft success.Previous study believed that clinica incorporated grafts had a continued locus minoris resistentiae, decisive therapeutic steps should be taken when the first indication of an inflammation occurs [30]. However, lateral packing or use of antibiotic ointments of graft affect the observation of postoperative early infection and graft change. Thus, technique of antibiotic ointment application to the lateral packing of graft following myringoplasty for preventing postoperative infections and improving the graft success was not sufficiently efficacious to justify routine use.

Apparently, mean ABG gain was not significantly different regardless of the use of antibiotic ointment or not. Also, similar with previous study [3, 10], no graft lateralization, significant blunting or medialization were found in any group during followup period. Although granular myringitis was seen in both groups, it could be well controlled by ofloxacin ear drops plus dexamethasone. In a word, there was no documented evidence of the no use of antibiotic ointment in the EAC affecting graft lateralization, significant blunting, and graft infection following myringoplasty.

These findings may challenge commonly held surgical beliefs about the need for lateral packing following myringoplasty. The advantages of this study is that no patient was lost to follow-up from each group, thus supporting the results. In addition, the graft change may be clearly observed at different followup points by endoscope because of the absence of EAC packing of biodegradable synthetic polyurethane foam. However, the limitations of this study was small sample size, possibility of bias due to lack of blinding, and short-term followup. In addition, bacterial culture and antimicrobial susceptibility testing were not performed for the patients with purulence discharge in both groups.

## Conclusions

Use and non-use of antibiotic ointments for lateral packing of graft are both comparable methods following tympanoplasty for postoperative infection and graft outcomes.

## Data Availability

The datasets supporting the conclusions of this article are included within the article.

## Abbreviations

EAC: External auditory canal
RCT: Randomized controlled trial
HRCT: High-resolution computed tomography
PTAs: Pure-tone averages
AC: Air conduction
BC: Bone conduction
ABG: Air bone gap
UAO: Use of antibiotic ointment
NAO: No use of antibiotic ointment
